# Evidence for parent-of-origin effects in autism spectrum disorder: a narrative review

**DOI:** 10.1007/s13353-022-00742-8

**Published:** 2023-01-30

**Authors:** Niamh M. Ryan, Elizabeth A. Heron

**Affiliations:** Neuropsychiatric Genetics Research Group, Department of Psychiatry, Trinity College Dublin, Dublin, Ireland

**Keywords:** Autism, Autism spectrum disorder, Imprinting, Mitochondrial DNA, Angelman, Prader-Willi

## Abstract

Autism spectrum disorder (ASD) is a heterogeneous group of early-onset neurodevelopmental disorders known to be highly heritable with a complex genetic architecture. Abnormal brain developmental trajectories that impact synaptic functioning, excitation-inhibition balance and brain connectivity are now understood to play a central role in ASD. Ongoing efforts to identify the genetic underpinnings still prove challenging, in part due to phenotypic and genetic heterogeneity.

This review focuses on parent-of-origin effects (POEs), where the phenotypic effect of an allele depends on its parental origin. POEs include genomic imprinting, transgenerational effects, mitochondrial DNA, sex chromosomes and mutational transmission bias. The motivation for investigating these mechanisms in ASD has been driven by their known impacts on early brain development and brain functioning, in particular for the most well-documented POE, genomic imprinting. Moreover, imprinting is implicated in syndromes such as Angelman and Prader-Willi, which frequently share comorbid symptoms with ASD. In addition to other regions in the genome, this comprehensive review highlights the 15q11-q13 and 7q chromosomal regions as well as the mitochondrial DNA as harbouring the majority of currently identified POEs in ASD.

## Introduction

Autism spectrum disorder (ASD) represents a heterogeneous group of common (prevalence of 0.83% (Vos et al. 2017)), early-onset, neurodevelopmental conditions (American Psychiatric Association 2013). ASD is characterised by differences in social interactions and communication as well as by restricted and repetitive behaviours and interests (Hodges et al. 2020). Much of the evidence to date points to the aetiology of ASD being related to brain development, converging on the abnormal development of synaptic functioning, excitation-inhibition balance and brain connectivity (Betancur et al. 2009; McFadden and Minshew 2013; Sohal and Rubenstein 2019).

ASD is known to be highly heritable (heritability approx. 80% (Bai et al. 2019)) with a complex genetic architecture. The majority of heritability is due to common variation, with rare and de novo structural variation contributing to a lesser extent (Searles Quick et al. 2021). ASD is typically grouped into either syndromic or non-syndromic ASD. Syndromic ASD, ASD plus or complex ASD occurs in approximately 25% of patients with the remaining 75% having what is referred to as essential or non-syndromic ASD (Carter and Scherer 2013). Syndromic ASD presents with additional phenotypes and/or dysmorphic features and is typically associated with chromosomal abnormalities or mutations in a single gene with diagnosis usually determined through some form of genetic testing (Fernandez and Scherer 2017; Sztainberg and Zoghbi 2016). For the majority of non-syndromic ASD cases, the genetic aetiology is unknown and is still proving challenging due to the considerable phenotypic and genetic heterogeneity present (Rylaarsdam and Guemez-Gamboa 2019). Other, more complex mechanisms of transmission may explain some of the as-yet unaccounted for heritability (Yoon et al. 2020). For instance, parent-of-origin effects (POEs) are a group of complex genetic effects that alter the phenotype in the offspring through a variety of mechanisms that modify gene expression in a parent-of-origin specific manner (Guilmatre and Sharp 2012). The most well-known mechanism responsible for POEs is genomic imprinting; however, other mechanisms include transgenerational effects (for example, maternal genetic effects and mother–offspring interaction effects), mitochondrial DNA, sex chromosomes and mutational transmission bias (for example, triplet-repeat-associated diseases) (see Guilmatre and Sharp 2012 for a general POE review). Advances in the field of ASD genomic research over the last 10 years, coupled with new and emerging evidence, make this an excellent time for this original review, highlighting the role of POEs in ASD aetiology. In this review, we introduce different POE mechanisms, giving motivation for their potential roles in ASD, and subsequently detail specific evidence for particular genes and genomic regions showing POEs in ASD. Throughout the review, we use the term autism spectrum disorder (ASD) but acknowledge that studies referred to may have had different definitions/diagnostic criteria and use the term autism for example.

## Parent of origin mechanisms

### Genomic imprinting

We inherit one copy of each autosomal gene from each of our parents. For the vast majority of these genes, both copies are capable of being expressed; however, for a small subset (128 genes in humans, geneimprint.com (imprint status = Imprinted-All), January 2022), one copy does not function (either partially or completely) due to the epigenetic mechanism, genomic imprinting. This mechanism silences one chromosome region in a parent-of-origin-dependent manner, leading to expression of only one copy of the gene. Imprinting is regulated by parental-specific epigenetic markers, including methylation and histone modifications established in gametogenesis and early embryonic development (Court et al. 2014; Hutter et al. 2010). Here we refer to imprinted genes as either maternally imprinted when the maternal copy of the allele is silenced or paternally imprinted when the paternal copy of the allele is silenced.

Imprinting is often tissue- and/or temporal-specific (Abramowitz and Bartolomei 2012; Reik and Walter 2001) and is necessary for optimal functioning of this small set of genes, most of which are involved in early development (including embryonic, placental and post-natal development), and behaviour, especially social behaviour (Falls et al. 1999; Lawson et al. 2013). Copy number variations (deletions or duplications), uniparental disomies (both chromosomes, or parts of a chromosome, are inherited from only one parent), aberrant methylation marks (epimutations) and point mutations can disrupt imprinting and have been identified as being associated with a range of human diseases and disorders, including neurodevelopmental and neuropsychiatric disorders (Soellner et al. 2017).

There are several motivations for considering imprinting in ASD. Early work on mouse chimaeras showed that imprinted genes have effects on general early growth, and on brain development in particular, including brain size and cell composition (Allen et al. 1995; Keverne et al. 1996), providing some of the first indications for imprinted genes having a potential role in neurodevelopment. Additionally, behavioural studies conducted on the adult stage of these mouse chimaeras pointed to imprinted genes in the brain as having an impact on behaviours such as aggression (Allen et al. 1995). In humans, many of the identified imprinted genes are expressed in the adult, influencing both cognitive and behavioural phenotypes (Isles and Wilkinson 2000; Davies et al. 2001, 2005)).

The most convincing hypothesis for the evolution of imprinting in mammals is the *genetic conflict* or *parental tug-of-war* hypothesis. This hypothesis suggests that maternally and paternally imprinted genes differentially regulate resources passed through the placenta from the mother to the developing embryo and subsequently exert an influence on the expression of traits in the offspring (Moore and Haig 1991). The conflict has advantages when females are likely to have more than one mate. In such a scenario, it is in the father’s genetic interest to maximise resources for the current pregnancy, as he cannot be certain if future offspring from this mother will share his DNA. In contrast, it is in the mother’s genetic interest to limit the resources shared with each offspring from each pregnancy, as they will all inherit half of her DNA. Thus, genes promoting growth are favoured by the father, whereas the mother will benefit from silencing her copy of such genes (Moore and Haig 1991). A good example of this is the maternally imprinted early growth-promoting gene *IGF2* (Constância et al. 2002). When maternal imprinting of *IGF2* is disrupted, there is an increase in birth weight, by up to 50% when both copies are expressed, resulting in the imprinting disorder Beckwith‐Wiedemann syndrome (Byars et al. 2014). Whereas when both copies are silenced, Silver-Russell syndrome results, which is characterised by undergrowth (Eggermann et al. 2010). In a study examining behavioural phenotypes, a higher proportion of children with Beckwith‐Wiedemann syndrome than expected also presented with ASD (6.8%) (Kent et al. 2008).

In the case of ASD, this parental tug-of-war hypothesis has been further extended to an imprinted brain developmental theory for ASD and schizophrenia. This theory proposes that imbalances during brain development (resulting from either enhanced effects of maternally imprinted genes, deficits in effects of paternally imprinted genes, or the action of both) can lead to ASD phenotypes. When the imbalance is in the opposite direction, with enhanced maternally biased effects, the risk of schizophrenia increases (Badcock and Crespi 2006, 2008; Byars et al. 2014; Crespi 2013; Crespi and Badcock 2008; Úbeda and Gardner 2010, 2011).

Syndromic forms of ASD have provided additional evidence for imprinted genes having a role in neurodevelopment. The syndromic forms of ASD, Angelman syndrome and Prader-Willi syndrome, are two well-characterised reciprocal chromosome 15q11-q13 imprinting disorders. These disorders present with mental, physical, cognitive and behavioural impacts on their phenotypes, and in a number of cases, with comorbid ASD (Hogart et al. 2010).

### Transgenerational effects: maternal genetic effects

Maternal genetic effects occur when the mother’s genotype exerts an influence on the offspring’s phenotype independent of the offspring’s genotype (Wolf and Wade 2009), for example, through the intrauterine environment. Several mechanisms, including the folate- and homocysteine-related pathways and immunity or inflammation, play critical roles in the development of the foetal nervous system in utero and can be impacted by maternal genetic effects. There is established and emerging evidence for their role in ASD (Azzini et al. 2020; Edmiston et al. 2017; Johnson 2003).

### Mutational transmission bias: trinucleotide repeats

Large expansions of specific trinucleotide repeat motifs result in trinucleotide-repeat-associated diseases. These motifs are often present in the general population at harmless levels but can expand to reach pathogenic levels when meiotic transmission to offspring takes place. As the rate of contraction and expansion during transmission is often different between males and females for many of these repeats, this results in POEs (Guilmatre and Sharp 2012). The neurodevelopmental disorder, Fragile X syndrome, is an X-linked dominant trinucleotide-repeat disorder and considered a syndromic form of ASD (Abbeduto et al. 2014).

### Mitochondrial DNA

Mitochondrial DNA (mtDNA), consisting of 37 genes, is exclusively maternally inherited and, together with many nuclear DNA genes, is responsible for generating the energy needed to power cells (Rossignol and Frye 2012). The complex role of mitochondria across all tissues and organs (only red blood cells have no mitochondria) means that mitochondrial disorders are often multi-systemic and multi-symptomatic. The number of mitochondria per cell varies widely (heteroplasmy), but the brain is known to have a very high demand on mitochondrial energy, in particular at neural synapses (Pei and Wallace 2018). If this energy supply is in any way disrupted, it can impact brain function and by extension could increase risk for neuropsychiatric disorders such as ASD (Chen et al. 2015; Giulivi et al. 2010; Pei and Wallace 2018; Yoo et al. 2017).

## Identified parent-of-origin effects in ASD

In the following sections, we detail specific chromosomes, genomic regions and genes where evidence of POEs in ASD has been identified. The key findings outlined here are also summarised in Table 1.

**Table 1:** Summary of the main POEs that have been identified as having an association with ASD. The location/gene, whether the effect is maternal or paternal, and the corresponding references are detailed

Location/gene	Maternal or paternal POE	References
Chromosome 7q		
7q21.1–22.2	Paternal identity-by-descent sharing	Lamb et al. (2005)
7q32.1–32.3	Maternal identity-by-descent sharing	Lamb et al. (2005)
7q32.3–34	Paternal identity-by-descent sharing	Ashley-Koch et al. (1999)
* CADPS2*	Maternal mono-allelic expression	Bonora et al. (2014)
* MEST*	Paternal transmission with maternal imprinting	Korvatska et al. (2011); Kwack et al. (2008); Brandler et al. (2018)
* CNTNAP2*	Maternal over-transmission vs paternal transmission	Arking et al. (2008)
Chromosome 15q11-13		
Chr15q11-13 Duplications	Maternal transmission	Cook et al. (1997); Depienne et al. (2009); Schroer et al. (1998); Urraca et al. (2013)
* UBE3A*	Maternal transmission with paternal imprinting	Urraca et al. (2013); Hsiao et al. (2019)
* SNRPN*	Paternal transmission with maternal imprinting; paternal transcript deficiency	Kim et al. (2008); Talkowski et al. (2012); Hogart et al. (2009)
X chromosome		
* FMR1*	Maternal transmission	Kaufmann et al. (2017)
Turner syndrome	Maternal transmission	Skuse et al. (1997); Good et al. (2003)
Mitochondrial DNA		
mtDNA point mutations	Maternal transmission	Wang et al. (2016); Graf et al. (2000); Pons et al. (2004); Connolly et al. (2010)
mtDNA haplogroups	Maternal transmission	Chalkia et al. (2017)
* MT-ND5* and *MT-ATP6*	Maternal transmission	Patowary et al. (2017)
Maternal genetic effects		
HLA-DR4 (chromosome 6p21.3)	Maternal genetic effect	Johnson et al. (2009)
GSTP1*A (chromosome 11q13.2)	Maternal genetic effect	Williams et al. (2007)
SHANK3 (chromosome 22)	Maternal genetic effect	Connolly et al. (2017)
RFC1 (chromosome 21)	Maternal genetic effect	James et al. (2010)

### Chromosome 7q

In a linkage study involving ASD-affected families and sib-pairs, significant evidence of paternal identity-by-descent sharing was identified on chromosome 7q32.3–34 (Ashley-Koch et al. 1999). In addition, the authors also detected significant linkage disequilibrium with paternal transmissions in multiplex and simplex ASD families. A genome-wide parent-of-origin linkage analysis conducted in affected sib-pairs identified two distinct regions, at 7q21.1–22.2 and at 7q32.1–32.3, as showing an excess of paternal and maternal identity-by-descent sharing in ASD, respectively (Lamb et al. 2005). Although neither of these peaks overlap with a known imprinted region, they lie close to imprinted gene clusters (Schanen 2006).

#### CADPS2 gene, chromosome 7q31.32

*CADPS2* shows tissue-specific mono-allelic expression (maternally inherited allele expressed in blood and specific brain regions; amygdala) (Bonora et al. 2014)*. Cadps2*-knockout mice show deficits in neuronal development and abnormal social behaviour (Sadakata et al. 2007, 2012; Sadakata and Furuichi 2009, 2010). Given its prominent role in the nervous system and the evidence from mouse studies, it has been considered an excellent candidate ASD gene, and evidence for downregulation in ASD compared to non-ASD brains has been shown (Voineagu et al. 2011). Bonora et al. (2014) identified a novel intragenic deletion in *CADPS2* in two siblings with mild intellectual disability (ID) and epilepsy (Bonora et al. 2014). In a follow-up mutation screening study in the same article, the authors discovered a missense variant of maternal origin that disrupts the interaction of *CADPS2* and the dopamine receptor D2DR, in a cohort of ASD/ID patients.

#### MEST gene, chromosome 7q32.2

*MEST* has long been known to be maternally imprinted with mono-allelic expression exclusively of the paternal copy (Pilvar et al. 2019). Maternal methylation appears to be driving this imprinting effect (Court et al. 2014; Partida et al. 2018; Schneider et al. 2012). Inactivation of the paternal allele for *MEST* in mice resulted in embryonic growth retardation and abnormal maternal behaviour suggesting a role in adult behaviour; additionally, methylation levels of *MEST* have been linked to cognitive ability (Lefebvre et al. 1998; Lorgen-Ritchie et al. 2019). Using whole-genome sequencing, an association was found between ASD and recurrent paternal rare cis-regulatory structural variants overlapping variant-intolerant genes (Brandler et al. 2018), including but not limited to *CNTN4*, *LEO1*, *RAF1* and *MEST*. Furthermore, these were transmitted to affected offspring more frequently than to their unaffected siblings. Positive association was found between *MEST* and ASD in a Korean male case–control cohort (Kwack et al. 2008), while a targeted sequencing study of the 7q32 region containing *MEST*, *COPG2* and *KLF14* showed nominal positive association (which did not survive Bonferroni correction) between two haploblocks and ASD in a study of 7q32-linked ASD families (Korvatska et al. 2011).

#### CNTNAP2 gene, chromosome 7q35-36.1

Using a combination of linkage and association analyses, two linkage peaks and a common genetic variant (displaying maternal over-transmission) significantly associated with ASD susceptibility were identified in the *CNTNAP2* gene (Arking et al. 2008). *CNTNAP2* is one of the largest genes in the human genome and is a member of the neurexin family, playing a role in brain development; regulating interactions between neurons and glia cells and contributing to the development of neuron axon structures (Waterhouse 2013). However, evidence for imprinting of *CNTNAP2* is mixed, some studies argue against *CNTNAP2* imprinting, at least in the adult human brain (Schneider et al. 2014), and others suggest imprinting might regulate expression of *CNTNAP2* under certain tissue-specific and/or developmental-stage specific conditions (I. S. Lee et al. 2015; Lin et al. 2012). A number of other studies have identified associations between this gene and ASD (in particular language development) but have either not tested for or have not identified a POE (Alarcón et al. 2008; Anney et al. 2012; Bakkaloglu et al. 2008; Chiocchetti et al. 2015; Li et al. 2010; Sampath et al. 2013; Whalley et al. 2011).

### Chromosome 15q11-13 region

#### Duplications of chromosome 15q11-13

Duplications in the 15q11-13 region (15q duplication syndrome, dup15q) are considered to be one of the most common cytogenetic abnormalities observed in ASD (1–3% of cases based on individual studies (Cook et al. 1997; Depienne et al. 2009), lower estimates of approximately 1 in 500 based on larger studies (Moreno-De-Luca et al. 2013)). The majority of duplications that result in an ASD phenotype are maternal in origin, with paternally derived duplications leading to either normal or mild phenotypes (for example, developmental delay) (Bolton et al. 2001; Browne et al. 1997; Cook et al. 1997; Mao et al. 2000; Schroer et al. 1998). Only duplications that specifically cover at least a portion of what is referred to as the Prader-Willi/Angelman syndrome critical region (15q11.2–13.1, BP2-BP3), approximately 5 Mb, are of interest (Finucane et al. 2016). Duplications not covering this region do not appear to have a clinical outcome (Browne et al. 1997; Chaste et al. 2014).

The majority of duplications (80%) result from a de novo maternal isodicentric chromosome 15 duplication (idic(15)), where an additional extra small chromosome is present, and ASD is highly likely for these individuals (Finucane et al. 2016; Moreno-De-Luca et al. 2013). The less frequent (20%) duplications occur within the long (q) arm of chromosome 15—maternal interstitial duplication 15 (int dup(15)), the majority of which are de novo (85%), with the remaining (15%) maternally inherited (Depienne et al. 2009; Finucane et al. 2016). These individuals are highly likely to present with ASD (Browne et al. 1997; Schroer et al. 1998; Urraca et al. 2013). The phenotype is similar to those with maternal idic(15) but often less severe, suggesting that there may be a maternal dosage effect for these duplications (Hogart et al. 2010; Moreno-De-Luca et al. 2013). Other research has suggested that maternal int dup(15) is not fully penetrant (Boyar et al. 2001), although it may be that penetrance is complete, but is presenting with a mild phenotype (Finucane et al. 2016).

The majority (65–75%) of Prader-Willi syndrome cases result from a loss of function of the paternal copy of maternally imprinted genes in the 15q11.2–13 region due to deletions, with the remainder of cases (20–30%) due to maternal uniparental disomy (mUPD) on chromosome 15 or imprinting defects (1–3%) (Cassidy et al. 2012). The clinical characteristics of Prader-Willi syndrome include infantile hypotonia and failure to thrive in infancy, hyperphagia and increased risk of obesity, mild-to-moderate intellectual disability and a distinctive behavioural phenotype (Cassidy et al. 2012; Dykens et al. 2011; Rangasamy et al. 2013). For individuals with Prader-Willi syndrome, there is an increased risk of ASD (comorbidity estimates between 12.3 and 36.5%, the lower rate from clinician determined diagnoses, the higher rate based on a screening tool) (Dykens et al. 2017; Veltman et al. 2005). Notably, a number of studies have shown that individuals with Prader-Willi syndrome as a result of a mUPD have a higher risk for ASD (Dykens et al. 2017; Schanen 2006; Veltman et al. 2004, 2005). This, coupled with the fact that maternally inherited duplications in the 15q11-13 region are among the most frequently observed chromosomal rearrangements in ASD, strongly suggests a role for the maternally expressed genes of this imprinted region being implicated in ASD (Dykens et al. 2017; Hogart et al. 2010; Schanen 2006).

#### UBE3A gene, chromosome 15q11.2

*UBE3A* plays a role in post-translational ubiquitination and regulation of synaptic plasticity (Greer et al. 2010) and is specifically paternally imprinted in mature neurons (Hsiao et al. 2019). A mouse model with three-fold increase in expression of maternal *Ube3a* in mature neurons showed impaired social behaviour and communication, with increased repetitive behaviour (Smith et al. 2011). Interestingly, the mouse model with two-fold increase in brain *Ube3a* had fewer behavioural deficits. These mice are good models for ASD showing that *Ube3a* is a dose sensitive gene.

In humans, even in the presence of a maternal duplication, *UBE3A* is still paternally imprinted and with higher expression levels (Herzing 2002). In a phenotype/genotype analysis of individuals with int dup(15), the authors concluded that the duplications were sufficient to give rise to the phenotype, most likely due to the over-expression of *UBE3A* (Urraca et al. 2013). Several linkage and association studies have examined this gene for links with ASD but with inconsistent findings (Cook et al. 1998; Guffanti et al. 2011; Nurmi et al. 2001, 2003).

The majority of Angelman syndrome cases are caused by deletions which lead to a decrease in expression of *UBE3A*, the remaining due to mutations in *UBE3A*, paternal uniparental disomy (pUPD) and imprinting defects (Dagli and Williams 2017). For Angelman syndrome, the clinical characteristics include motor dysfunction, intellectual disability, speech impairment and seizures (Greer et al. 2010; Rangasamy et al. 2013). High rates of comorbidity with ASD have been shown for Angelman syndrome (Mertz et al. 2014).

As mentioned, Prader-Willi syndrome individuals with a mUPD show more ASD traits than Prader-Willi syndrome individuals with paternal deletions. Using human post-mortem brain samples, cortical tissue expression of *UBE3A* was shown to be substantially higher for Prader-Willi syndrome individuals with mUPD than for those with a deletion form (Hogart et al. 2007).

Iossifov et al. reported a de novo T485A missense mutation in *UBE3A* in an ASD male proband; however, they did not test whether the variant was present on the paternal or maternal copy of the chromosome (Iossifov et al. 2014). A functional investigation of this mutation in *UBE3A* in human cell culture experiments showed that the T485A variant inhibits *UBE3A* self-regulation, leading to increased *UBE3A* activity, and increases dendritic spine formation (Yi et al. 2015).

#### SNRPN gene, chromosome 15q11.2

*SNRPN* is a maternally imprinted gene that regulates expression of Nr4a1, a nuclear receptor critical for cortical neuron development (Barr et al. 1995; H. Li et al. 2016; Reed and Leff 1994). Several studies have reported uniparental methylation POEs for this gene (Court et al. 2014; Partida et al. 2018). *SNRPN* has been proposed as a likely candidate gene for Prader-Willi syndrome (Cassidy et al. 2000). In a trio study design, two SNPs in *SNRPN* showed marginal imprinting effects (Kim et al. 2008) and a balanced chromosomal abnormality was identified in an individual with ASD without Angelman syndrome or Prader-Willi syndrome (Talkowski et al. 2012). Through a post-mortem brain tissue analysis, Hogart et al. 2009 identified deficiencies in the paternally expressed transcripts of *SNRPN* in a female individual with ASD and milder Prader-Willi-like characteristics (Hogart et al. 2009). In addition, methylation levels of *SNRPN* have been linked to cognitive ability (Lorgen-Ritchie et al. 2019).

### FMR1 gene, X chromosome

Fragile X syndrome is predominantly caused by a CGG triplet repeat mutation expansion in the promoter region of the *FMR1* gene at the chromosome Xq27.3 locus (Kaufmann et al. 2017). This syndrome is understood to be the most common single-gene cause of ASD (1–6% of ASD cases) (Kaufmann et al. 2017). As Fragile X syndrome is X-linked, it affects more males than females and more severely (Kaufmann et al. 2017). Fragile X syndrome is characterised by males almost always exhibiting moderate intellectual disability, having a characteristic appearance (macrocephaly with a prominent forehead, long face, large protruding ears and a prominent chin, the dysmorphism is milder in females) and behaviour (Fernandez and Scherer 2017; Sherman et al. 2005). Males carrying a full mutation (> 200 repeats) do not produce offspring, whereas males with an intermediate (45–54 repeats) or premutation (45–54 repeats) will pass it on, but only daughters will inherit the mutation (Nolin et al. 2019). For maternal transmissions, pre-mutations can expand to full mutations, which is rarely the case for paternal transmissions (Nolin et al. 2003, 2019). This results in a greater chance of offspring developing Fragile X syndrome when the mother is the premutation carrier compared to the father. In addition, there is a paternal bias for contraction of the premutation triplet repeats (Nolin et al. 2019). This triplet repeat mutation results in abnormal methylation of *FMR1* and either partial or complete silencing of the gene (Kidd et al. 2019). The upshot is decreased production of the Fragile X mental retardation protein (FMRP) which has a vital role in synaptic plasticity and brain development as it regulates protein synthesis at the synapses (Penagarikano et al. 2007).

### Turner syndrome, X chromosome

Turner syndrome results when one of the X chromosomes (maternal or paternal) is either partially or fully missing and shows an increased rate of ASD (Creswell and Skuse 1999; Donnelly et al. 2000; Skuse et al. 1997). Turner syndrome clinical characteristics include growth disorders, reproductive system and cardiovascular abnormalities and autoimmune diseases (Cui et al. 2018). From a POE perspective, the phenotype varies depending on whether the single X chromosome has been maternally or paternally inherited (Donnelly et al. 2000; Skuse et al. 1997). Turner syndrome girls with a paternally derived X chromosome were shown to be more socially adept than girls with a maternally derived chromosome (Skuse et al. 1997). This led to Skuse et al. (1997) hypothesising the presence of a maternally imprinted genetic locus for social cognition on the X chromosome, which was further supported by Good et al. (Good et al. 2003; Skuse et al. 1997). Given the higher prevalence of ASD in males, and as they do not inherit the paternal X chromosome, Skuse has also speculated that a paternally expressed locus may be present on the X chromosome that gives a protective effect against ASD (Skuse 2000).

### Mitochondrial DNA mutations

While mtDNA mutations are mainly associated with classical mitochondrial diseases (MELAS syndrome, MERRF syndrome, Leigh syndrome, Leber’s hereditary optic neuropathy, etc.), the majority of these diseases have some form of neurological, neurodevelopmental or psychiatric component, not surprising considering the energy demand of both the central nervous system and the brain (Bressan and Kramer 2021; C. S. Dela Cruz and Kang 2018). Studies of biochemical markers of abnormal aerobic respiration, such as elevated lactate levels, have provided indirect evidence of mitochondrial dysfunction in ASD (Correia et al. 2006; Žigman et al. 2021). Cohorts of individuals with mitochondrial disease have also been shown to have increased risk of ASD in comparison to the general population (10–20% rates of ASD compared to 2% in the general population), and the reverse is also true; individuals with ASD have been shown to have increased rates of mitochondrial disease compared to the general population (1–5% rates of mitochondrial disease compared to 0.05% in the general population) (Legido et al. 2013). Together, these findings suggest a real relationship between ASD and mitochondrial diseases. Interestingly, some imprinted genes also appear to affect mitochondrial function (Bressan and Kramer 2021; Panov et al. 2020; Urraca et al. 2013; Victor et al. 2021; Yazdi et al. 2013).

A systematic review by Cruz et al. (2019) identified a number of studies showing that genetic variations in mtDNA are associated with neurological disorders, including neurodevelopmental disorders such as ASD. A whole-exome sequencing study of ASD-affected individuals, together with their unaffected siblings and mothers, showed that the transmission of mtDNA point mutations of suspected high pathogenicity was greater between mothers and affected children than between mothers and unaffected siblings, with a higher frequency of these mutations in the ASD probands with lower Intelligence quotient (IQ) and/or deficit in social behaviour (Wang et al. 2016). Two mtDNA genes, *MT-ND5* and *MT-ATP6*, have been linked to ASD through a whole-exome sequencing study of ten multiplex families (Patowary et al. 2017). This builds on other research that identified mutations in the *MT-ATP6* gene as linked to ASD (Piryaei et al. 2012; Rossi et al. 2017). A mutation of the *MT-TK* gene was found to be associated with members of a family (mother and three children) with multiple neurological disorders, including a boy with an autism-like phenotype and was suggested as the basis for his ASD (Graf et al. 2000). For the *MT-TL1* mtDNA gene (associated with mitochondrial encephalopathy, lactic acidosis and stroke-like episodes (MELAS) syndrome which can coexist with ASD (Griffiths and Levy 2017)), two mutations have been potentially linked to ASD: A3243G in five mother–offspring pairs (Pons et al. 2004) and A3260G in a single family (Connolly et al. 2010). In an alternative approach, Chalkia et al. examined mitochondrial lineages (ASD-affected individuals, their parents and siblings) which encompass ancient mtDNA functional polymorphisms for association with ASD risk (Chalkia et al. 2017). They found evidence that particular European, Asian and Native American haplogroups showed a significant increase in risk for ASD when compared with the most common European haplogroup.

### Maternal genetic effects

Johnson et al. identified a maternal genetic effect in mothers of ASD offspring for the *HLA-DR4* (chromosome 6p21.3) allele, suggesting that this finding supports the possibility of an immune component to ASD acting during pregnancy (Johnson et al. 2009). An over-transmission of the GSTP1*A haplotype on chromosome 11q13.2 to mothers of ASD offspring was identified by Williams et al. 2007 (Williams et al. 2007). *GSTP1* is associated with oxidative stress, furthering the evidence for inflammation in the intrauterine environment impacting on ASD risk. *SHANK3* (chromosome 22q13.33) encodes a synaptic scaffold protein, essential in the postsynaptic density, and several studies have found associations with ASD (Boccuto et al. 2013; Durand et al. 2007; Leblond et al. 2014; Sanders et al. 2015). Connolly et al. presented evidence to suggest that a mutation in the mother’s *SHANK3* gene could increase the likelihood of her offspring having ASD (Connolly et al. 2017). The folate pathway supports the change between cell proliferation and differentiation during the early stages of development. If the availability of folate derivatives in the intrauterine environment is altered, neurodevelopment can be disrupted (James et al. 2010). Through a case–control analysis followed by a trio design, James et al. found that the maternal genotype carrying a functional polymorphism in *RFC1* (involved in folate metabolism, chromosome 21) was associated with an increased risk of ASD, whereas the offspring genotype was not (James et al. 2010).

## Discussion

The aetiology of ASD is driven by a combination of environmental and genetic factors (Searles Quick et al. 2021), though a large proportion of the genetic risk remains unexplained. To improve our understanding of ASD pathogenesis, we need to investigate alternative models of inheritance. POEs are good examples of such alternative forms that should be pursued to help explain some of the missing heritability of this complex disorder. Despite this, it remains an under-studied branch of ASD research.

In this review, we have described different mechanisms of POEs and presented a range of evidence for their role in ASD. We have summarised evidence supporting both maternal and paternal imprinting at the 7q32.1–32.3 and 15q11-13 chromosomal regions, related to both ASD and the syndromic forms of ASD, Angelman and Prader-Willi syndromes, showing how crucial the imprinted brain is for the developmental and behaviour phenotypes associated with ASD. Indeed, a recent study has shown that both imprinting and brain development correlate with ASD (Li et al. 2020). There is also increasing evidence, both from genetic and biochemical studies, suggesting that genetic variants in mitochondrial DNA are associated with ASD. While the relationship between ASD and mitochondrial diseases is clear, future genetic studies that include mitochondrial genetic variants (which are historically often excluded from the analysis of genome-wide association studies and next-generation sequencing studies) will be needed to assess the full impact of mitochondrial variants in ASD probands and the developing autistic brain.

We have aimed to present POEs where there is a reasonable degree of evidence and have not included studies where there is uncertainty regarding the presence of a POE. However, it should be noted that many of the ASD POE findings detailed in this review, in particular the earlier studies, are based on small sample size datasets, often individual families or a small number of cases. This is further complicated by the fact that ASD is a heterogeneous phenotype. Therefore, comparing studies can be problematic due to different diagnostic criteria (e.g., screeners versus diagnostic tools), potentially increasing phenotypic heterogeneity. In addition, the findings that have been identified are often understood to be rare. As with all rare variant studies, this raises questions about power for statistical hypothesis testing (Wray and Gratten 2018); however, increasing sample size and/or replicating in many scenarios is not feasible. The recent generation of whole genome and whole-exome sequencing data from large ASD cohorts, such as the studies by Iossifov et al. and Brandler et al., provides an even greater opportunity to identify rare ASD variants which may have parent-of-origin effects; however, care must be taken to ensure the right statistical tests are performed to identify POEs in these studies (Brandler et al. 2018; S. Connolly and Heron 2014; Iossifov et al. 2014).

We see in this review that POEs play a role in several syndromic forms of ASD (Angelman, Prader-Willi and Fragile X syndromes). Although syndromic forms account for only a small proportion of ASD cases (~ 5%), the biological insights gained from studying syndromic ASD may offer avenues for the understanding of non-syndromic ASD (Sztainberg and Zoghbi 2016). However, care needs to be taken with identifying ASD comorbidity, as syndromic forms of ASD often have different developmental trajectories from non-syndromic ASD (Sztainberg and Zoghbi 2016). Furthermore, care needs to be applied in the choice of ASD diagnostic tool, as diagnosing ASD when the mental age is low is difficult with standard tools (Lord et al. 2000; Miller et al. 2019). In the case of Angelman syndrome for example, Hogart et al. note that the comorbidity studies should be interpreted with caution due to the severity of the cognitive and language impairments and the low mental age range which could be resulting in an over-estimate of ASD comorbidity in Angelman syndrome (Hogart et al. 2010; Trillingsgaard and Østergaard 2004). The assumption here is also that the same set of genes identified in these syndromes are also involved in the comorbid ASD. When IQ or mental age is low, there is the possibility that the ID could be mimicking aspects of the ASD phenotype (Grafodatskaya et al. 2010).

As is clear from the evidence presented here, determining POEs in ASD is an evolving field. For example, with regard to imprinting, it is not a simple case of taking a list of imprinted genes and determining if ASD is linked or associated with these genes. Rather, the imprinting status for a number of these genes is also an evolving area of research. The temporal- and tissue-specific nature of imprinting poses challenges for determining whether or not a gene is imprinted. Authors have also noted and demonstrated that imprinting and maternal genetic effects are known to mimic each other, making determination of the POE type more difficult (Connolly and Heron 2014; Wolf and Wade 2009).

To conclude, the evidence reviewed here converges on the role of ASD POEs in brain development and brain functioning. In particular, a number of the POEs show involvement with synaptic functioning (for example, mutations in *FMR1, UBE3A, SHANK3*, mtDNA). This is an exciting and expanding field of research, linking together what, on the surface, appear to be quite disparate mechanisms. It is curious that such distinct mechanisms all appear to converge on similar developmental and functional pathways, leading to similar etiological outcomes/presentations. Given the early stages of our understanding of POEs in ASD, we are yet to fully feel their implications in the clinical setting. No doubt as genomic technologies develop, including, for example, technologies that allow for epigenetic profiling, diagnostic testing for POEs will become more straightforward which has the potential to impact diagnostic testing in ASD. In addition, POEs offer another avenue to further tease apart the complex nature of the ASD phenotype with the ultimate goal being to identify usable drug targets and biomarkers that will have tangible impacts for ASD in terms of treatments and interventions. One current example of this is Nr4a1, which has been proposed as a possible drug target for *SNRPN*-related neurodevelopment disorders, including Prader-Willi syndrome and ASD (H. Li et al. 2016) and is already under consideration as a drug target in cancer (Hedrick et al. 2015; S. O. Lee et al. 2014). We are interested to see how the field develops in the future and how our understanding of ASD and related disorders will change with a better understanding of the contributions of POEs to ASD.

## Data Availability

As this is a review article no data were analysed.
